# Supplementary material for: How ligands regulate the binding of PARP1 with DNA: Deciphering the mechanism at the molecular level
Source: PLoS One. 2023 Aug 15;18(8):e0290176. doi: 10.1371/journal.pone.0290176 (PMC10426920; doi:10.1371/journal.pone.0290176)
Supplement: S1 File — (DOCX) [file pone.0290176.s001.docx]

**How ligands regulate the binding of PARP1 with DNA: deciphering the mechanism at the molecular level**

**Supporting information.**


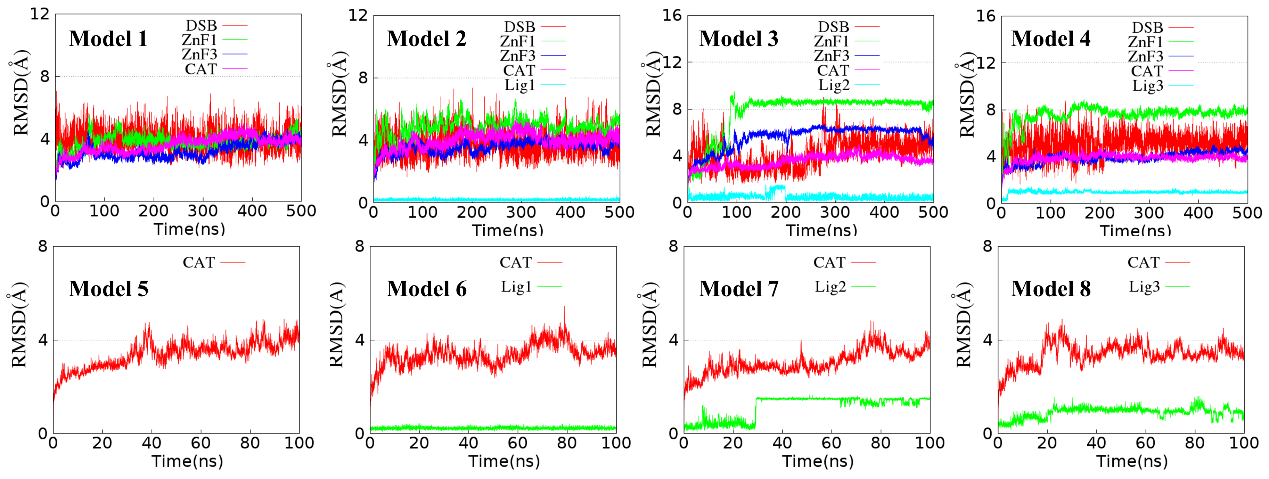


**Fig S1.** RMSDs of all system in Table 1.


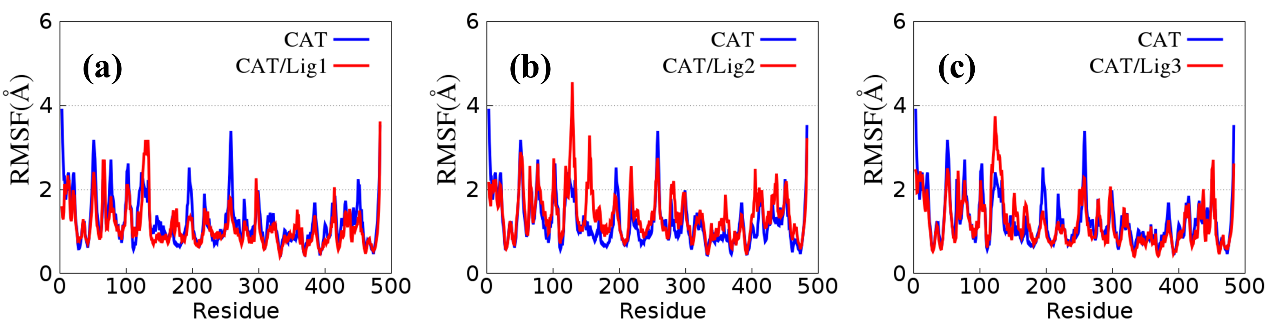


**Fig S2.** RMSFs of Model 6 to Model 8 with CAT monomer.


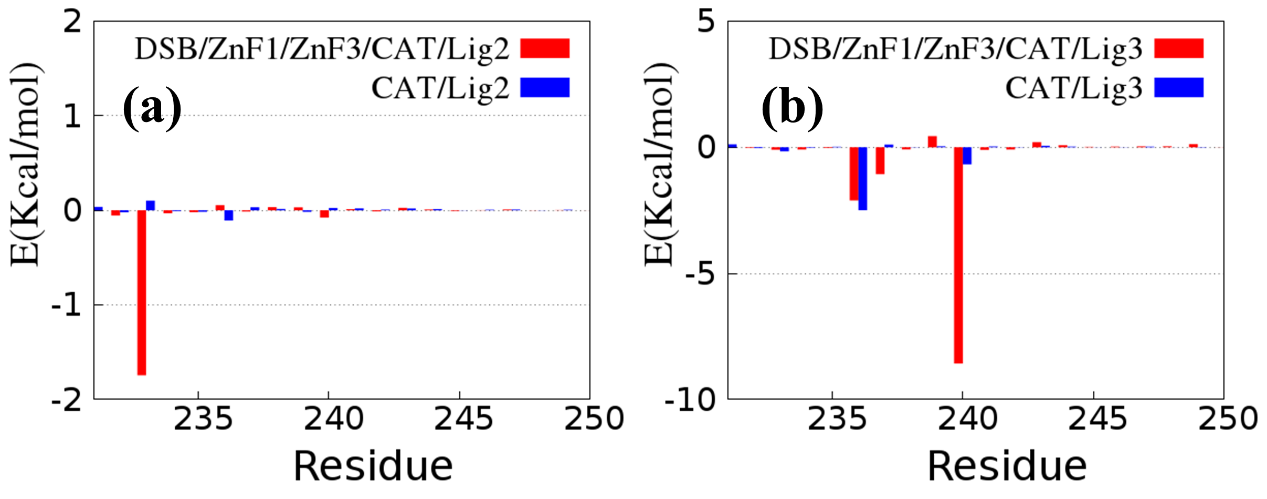


**Fig S3.** (a) Per-residue energy contribution spectra of CAT on the surface of CAT/ZnF1. (b) Per-residue energy contribution spectra of CAT on the surface of CAT/ZnF3. Red, blue, green, and yellow bar represent Lig1, Lig2, Lig3 and without ligand binding to CAT.


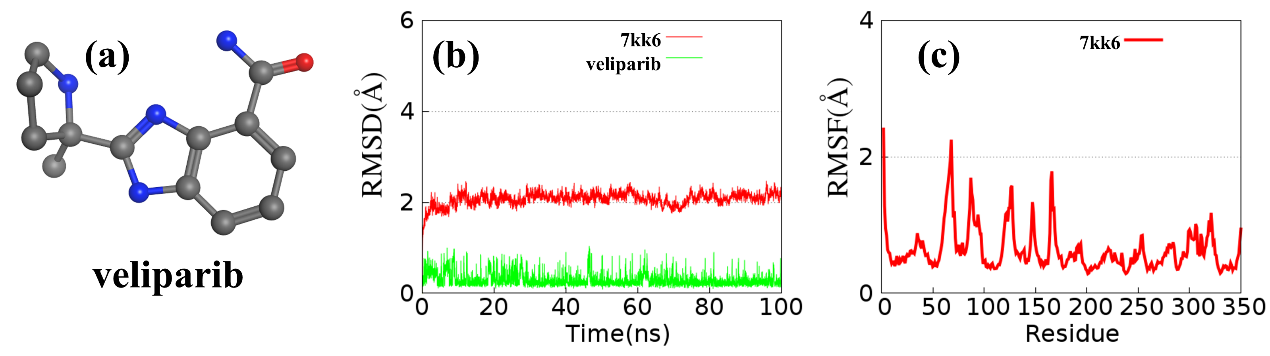


**Fig S4.** (a) 3D-structure of veliparib. (b) RMSD of 7kk6 which contains the interaction between CAT and veliparib. (c) RMSF of CAT in 7kk6.
