# Supplementary material for: How ligands regulate the binding of PARP1 with DNA: Deciphering the mechanism at the molecular level
Source: PLoS One. 2023 Aug 15;18(8):e0290176. doi: 10.1371/journal.pone.0290176 (PMC10426920; doi:10.1371/journal.pone.0290176)
Supplement: Attachment — Submitted filename: PONE-D-23-05051-Reviewer-report.pdf [file pone.0290176.s002.pdf]

Manuscript ID: PONE-D-23-05051

Recommendation: Major revision is required

The manuscript entitled “How ligands regulate the binding of PARP1 with DNA: deciphering the mechanism at the molecular level" by Kai Wang and coworkers explored the structural and dynamic conformational changes in multi-domains of PARP1 induced by ligands. The molecular dynamics calculations have been systematically performed and the results were analyzed extensively and correlated well with the energy decomposition method. Overall, this work sounds good and interesting to the readers.

However, the following points need to be addressed before publishing this manuscript.

1. Authors should provide the common name or class of the ligands and the IUPAC nomenclature of ligands.
2. The figure captions and figures are given in different places, it troubles a lot the reviewer to understand the results. It is highly inconvenient to critically analyze the results.
3. In all the figures, Kcal/mol is given wrongly instead of kcal/mol
4. In Figure 6, is there any label for the x, y, and Z axes? Are there any noticeable differences? What is the meaning of the different colors? It would have been given in the figure captions.
5. In conclusion, the authors should address, predicting the functional mechanism of PARP1 and the new directions to develop PARP1 inhibitors.

Other minor corrections:

1. In figure 4f, the Y axis label is partially hidden. It should be taken care of.
2. In materials and methods, in the MD simulation part, 1000 kcal/(mol·Å) - should be corrected.
3. In Table 2, Kcal/mol - should be corrected
4. In the whole manuscript, Kcal/mol – this correction should be carried out
