# Supplementary material for: How ligands regulate the binding of PARP1 with DNA: Deciphering the mechanism at the molecular level
Source: PLoS One. 2023 Aug 15;18(8):e0290176. doi: 10.1371/journal.pone.0290176 (PMC10426920; doi:10.1371/journal.pone.0290176)
Supplement: Attachment — Submitted filename: point-to-point-reviewer1.docx [file pone.0290176.s003.docx]

Thank you a lot for your kind and careful reviewing. We greatly appreciate your valuable comments and suggestions, which helped us to improve the technical quality and presentation of our manuscript. All your comments have been addressed and the manuscript was revised accordingly. All modifications were highlighted in red in the revised manuscript. In addition, **extensive English editing was carried out to improve the English expression of this paper**. In the following, we give a point-by-point reply to your comments.

**Reply to the reviewer 1**

Comments to the Author

The manuscript entitled “How ligands regulate the binding of PARP1 with DNA: deciphering the mechanism at the molecular level” by Wang et al, aimed to identify the mechanism and the effect of the inhibitor molecules on PARP1 binding to DNA by molecular dynamics simulation approach.

The work is not well-written. Some sentences need an improvement of the English language.

As example:

“To facilitate the development of potential inhibitors of PARP1 through structure-based computational design, it is of great significance to clarify the differences in structural dynamics and key residues between CAT/inhibitors and DNA/PARP1/inhibitors.” This sentence should be changed as “To facilitate the development of potential inhibitors of PARP1, it is of great significance to clarify the differences in structural dynamics and key residues between CAT/inhibitors and DNA/PARP1/inhibitors through structure-based computational design.”

**Response:** We greatly appreciate the reviewer’s valuable comments and suggestions. Extensive English editing was carried out to improve the English expression of this manuscript.

**Others concerns:**

1.The Abstract mentioned “Furthermore, the interaction between ligands and catalytic binding sites can be transferred to the DNA recognition domain of PARP1 by a strong negative correlation movement among multi-domains of PARP1.”

But Figure 6 only explores the linear correlation between all CAT residues, that is, the interactions within the CAT residue domain (catalytic site domain). There is a strong negative correlation motion within CAT, and the combination of DSB and PARP1 causes changes in the CAT domain correlation. The conclusion should be more comprehensive. For example, the interaction between ligands and catalytic binding sites can affect DNA recognition domains, The combination of DSB and PARP1 also affects the conformation of CAT. Additionally, how can we determine if this negative correlation has transferred to the domain of DNA recognition? The author needs to clarify.

**Response:** Thank you for pointing out this ambiguous description. We really appreciate it. Following your comment, we added some content to describe the correlation and interaction among ligands, CAT, ZnF1 and DSB more comprehensively in the revised manuscript. In addition, possible mechanisms for the negative correlation transmission were discussed in the revised manuscript.

The content added to the **Correlation analysis for CAT** section of the revised manuscript is as follows:

“Possible transmission mechanisms deserve to be discussed. The RMSD of ZnF1/ZnF3/CAT/DSB between any two of the three systems (ZnF1/ZnF3/CAT/DSB/Lig1, ZnF1/ZnF3/CAT/DSB/Lig2, and ZnF1/ZnF3/CAT/DSB/Lig3) is below 0.1Å, which indicates that the ZnF1/ZnF3/CAT/DSB is nearly identical in the three systems if the interaction with ligands is not considered. Therefore, it is obvious that the observed negative correlation is induced by inhibitors. The catalytic binding site of CAT and the interaction interface of CAT, DBD and DSB are located at two different regions of CAT, far away from each other, as shown in Fig 4d. The two regions are connected by residues. The binding energies of ligand/CAT, ZnF1/CAT, and DSB/CAT are about -30kcal/mol, which implies that their interactions are stable. Consequently, it is inferred that the interaction between ligands and CAT can be transferred to the interaction between CAT and DSB by the negative correlation of domains in CAT.”

The content added to the **Conclusions** section of the revised manuscript is as follows:

“Secondly, ligands can indirectly influence the binding stability of CAT/DSB and ZnF1/DSB. Specifically, Lig2 enhances the binding stability between CAT and ZnF1. Lig3 enhances the interaction between CAT and ZnF3. Lig1 strengthens the interaction between ZnF1 and ZnF3.”

In addition to explaining the intra domain correlation between DSB/Znf1/Znf3/CAT and monomer CAT when combined with Lig1, Lig2, and Lig3, it is also necessary to compare and explain the differences in intra domain correlation between DSB/Znf1/Znf3/CAT and monomer CAT (longitudinal comparison).

Figure 6 shows that in the DSB/Znf1/Znf3/CAT system, Lig1 greatly enhances the negative correlation between WGR and HD, while Lig2 and Lig3 weaken the negative correlation. However, in the monomer CAT system, the binding of Lig1 does not have a significant or even reduced effect on the correlation; In monomer CAT, Lig2 enhances the negative correlation between HD and ART, while in the DSB/Znf1/Znf3/CAT system, Lig2 reduces the negative correlation and should be discussed separately.

**Response:** Thanks for your valuable comments and suggestions. We really appreciate it. As suggested by the reviewer, we have added in-depth explanations and discussions to compare and explain the differences in intra domain correlation between DSB/Znf1/Znf3/CAT and monomer CAT. And meanwhile, some content was added to discuss the different mechanisms of ZnF1, ZnF3, and DSB on the binding process of CAT.

The content added to **Correlation analysis for CAT** section of the revised manuscript is as follows:

“In addition, it is also necessary to compare and clarify the differences in intra domain correlation between DSB/Znf1/Znf3/CAT and monomer CAT. Firstly, the apo-states of DSB/Znf1/Znf3/CAT and monomer CAT were compared. The correlation among WGR, HD and ART in these two systems shows no significant difference, which indicates that the interaction of CAT/Ligand rather than that of CAT/DSB and CAT/ZnF1 affects the intra domain correlation of CAT. Secondly, DSB/Znf1/Znf3/CAT/Ligand and CAT/Ligand were compared. The correlation of CAT between monomer CAT and complexes exhibits significant differences. For example, compared to the CAT/Lig1 system, Lig1 in DSB/Znf1/Znf3/CAT/Lig1 greatly enhances the negative correlation between WGR and HD, while Lig2 and Lig3 slightly weaken the positive correlation.”

The content added to the **Conclusions** section of the revised manuscript is as follows:

“PARP1 inhibitors can bind to catalytic binding site of PARP1 and thereby affect the motion correlation among the residues of CAT. Based on the correlation, PARP1 inhibitors indirectly affect the interaction between CAT and DNA recognition regions as well as DNA, ultimately inhibiting the DNA recognition and repair functions of PARP1.”

2. “On the other hand, it is not reliable to use the binding energy between the CAT domain and the ligand as a measure of ligand activity, because it may in some cases differ greatly from the binding energy between the ligand and the PARP1/DNA complex. However, for PARP1/DNA/ligand, the stronger the binding stability between the ligand and PARP1, the stronger the binding stability between PARP1 and DNA.”

What is the significance of studying CAT/ligands? Isn't it enough to only study PARP1/DNA/ligands with practical significance?

**Response:** Thank you for your valuable comment. We really appreciate it. Currently, it is impossible to only select PARP1/DNA/ligands for binding mechanism research and further inhibitor design. Firstly, since PARP1 contains multiple domains, the complete structure of PARP1 has huge conformational space, with many possibilities for its functional conformation. For simplicity and efficiency, CAT monomer or CAT/Ligand are commonly used as the target for designing PARP1 inhibitors. Complete and accurate crystal structure of PARP1 is currently unavailable. Although computational methods can be used to predict the complete PARP1 structure, the predicted structure requires further structural optimization of MD simulation and experimental verification. Therefore, predicting complete structure of PARP1 with structural optimization of MD simulation and developing new scoring function to compensate for the limitations of experimental methods in PARP1 crystallization can facilitate the development of potential PARP1 inhibitors. Finally, the PARP1 structure used in this work contains most of the structural domains of PARP1. And meanwhile, MD simulations and binding energy calculation were used to describe the binding mechanism among ligands, PARP1 and DSB. The strategy employed in this work is highly potential in obtaining reasonable PARP1 structure and useful for further research on molecular docking and inhibitor design.

The content added to **Binding mechanisms among ligands, PARP1 and DSB** section is as follows:

“Finally, since PARP1 contains multiple domains, the complete structure of PARP1 has huge conformational space, with many possibilities for its functional conformation.

For simplicity and efficiency, CAT monomer or CAT/Ligand are commonly used as the target for designing PARP1 inhibitors. Complete and accurate crystal structure of PARP1 is currently unavailable. Although computational methods can be used to predict the complete PARP1 structure, the predicted structure requires further structural optimization of MD simulation and experimental verification. The PARP1 structure used in this work contains most of the structural domains of PARP1. By introducing DSB and DBD domains, potential binding mechanisms for ligand regulation of PARP1 function were discovered. As shown in Fig 7d and Table 7, the enhancement in binding stability between ligands and CAT can strengthen the binding stability of CAT with DSB.”

3. The pictures are generally not clear enough.

The RMSF measurement of residual flexibility shown in Figure 2a of Figure 2 shows that the presence or absence of ligands can affect the flexibility of the ZnF1 structure, especially around the last 20 residues. The binding of ligands clearly increases the atomic fluctuations of the complex.

**Response:** Thank you for your careful reviewing. We really appreciate it. For the ZnF1 domain, the last 20 residues construct discrete loop structure which is far away from the center of ZnF1, as shown in the area protruding from the bottom left corner of Figure 2b. These residues of ZnF1 are far away from the functional interface of ZnF1/DSB and ZnF1/CAT. In addition, these residues not only have significant structural flexibility, but also have a certain degree of randomness. To ensure the completeness of the original crystal structure, we retained this structure in molecular dynamics simulation, but data analysis was not conducted on this flexible area. Therefore, the difference in structural flexibility in this area does not originate from ligands and does not need to be studied.

In Figure 2b, the interactions between Met38 and Phe39 and these structural domains should be depicted separately, and it is necessary to clarify how the residues on the interface between ZnF1 and DSB and CAT are determined.

**Response:** Thank you for your careful reviewing. We really appreciate it. Following your comment, we have modified the colors of Met38 and Phe39 in the revised manuscript to better show the interaction interface between ZnF1, DSB, and CAT. In addition, how to determine the residues on the interface between ZnF1, DSB, and CAT was explained in the revised manuscript.

The content added to **Conformational changes in ZnF1** section is as follows:

“The interaction interface among ZnF1, DSB, and CAT was determined based on the distance between interacted atoms in the crystal structure of 4OPX. The specific steps are as follows: selecting a range of 4 angstroms among the atoms of residues or nucleotides, connecting these discontinuous residues together by other connected residues, and defining them as the interaction interface.”

Figures 2c and d are not clear enough. The article mentions the enhancement of A9 binding force, but they are not specifically labeled in Figures c and d. Specific amino acids need to be specifically labeled.

**Response:** Thank you for your careful reviewing. We really appreciate it. Following your comment, A9 was labeled in the latest Figure 2c and the key residues in Figure 2d were labeled accordingly.

Similarly, what is the basis for determining the interface residues between ZnF3/DSB/CAT in Figure 3b? How are overlapping residues determined?

**Response:** Thank you for your careful reviewing. We really appreciate it. The basis for determining the interface residues between ZnF3/DSB/CAT in Figure 3b is similar to that for ZnF1/DSB/CAT. These overlapping residues determined by the residues in the defined overlap region depending on their proximity to other interacting residues or nucleotides.

R345 is not indicated in Figure 5df.

**Response:** Thank you very much for your careful reviewing. It should be R348. The mistake was corrected in the revised manuscript.

Figure 5g-i should not be the last sampling structure of MD simulation track to display the change of ligand binding mode, but should use clustering method to find representative conformation to display the change of ligand binding mode.

**Response:** Thank you for your suggestion. We really appreciate it. Clustering method is indeed one of the best methods for comparing macromolecular conformational changes. The method employed in this work conforms to the clustering method based on RMSD. The method is used to describe the change in the binding sites of the ligand structure which contains both translational and rotational components. It is found in this work that Lig 2 undergoes a certain degree of translation and rotation compared to the crystal structure, leading to changes in the key functional residues. What’s more, it is very convenient to use the clustering method based on RMSD for the purpose of structural comparison. As shown in Fig S1, the RMSD value is very stable at the late stage of MD simulation. Therefore, structures obtained from the late stage of MD simulation can be used to display the translational and rotational differences of ligand between stable MD simulation and crystal structure.
