# Supplementary material for: How ligands regulate the binding of PARP1 with DNA: Deciphering the mechanism at the molecular level
Source: PLoS One. 2023 Aug 15;18(8):e0290176. doi: 10.1371/journal.pone.0290176 (PMC10426920; doi:10.1371/journal.pone.0290176)
Supplement: Attachment — Submitted filename: Response to Reviewers.docx [file pone.0290176.s004.docx]

Thank you a lot for your kind and careful reviewing. We greatly appreciate your valuable comments and suggestions, which helped us to improve the technical quality and presentation of our manuscript. All modifications were highlighted in red in the revised manuscript. In the following, we give a point-by-point reply to your comments.

**Reply to the reviewer #1**

Comments to the Author

The manuscript entitled “How ligands regulate the binding of PARP1 with DNA: deciphering the mechanism at the molecular level” by Wang et al, aimed to identify the mechanism and the effect of the inhibitor molecules on PARP1 binding to DNA by molecular dynamics simulation approach.

The present study is logic and well designed, the analyses have been properly conducted, and the conclusions can be supported by their results. To further improve this study, there are some minor comments:

**Response:** We appreciate the reviewer’s valuable comments and suggestions.

“Fig 5d-f describes the per-residue energy contribution of CAT induced by the ligands. Among them, the repulsion of K363 and R345 in CAT significantly increases in the systems of DSB/ZnF1/ZnF3/CAT/Lig1 and DSB/ZnF1/ZnF3/CAT/Lig3.” It should be R348. The mistake has not been corrected yet in the revised manuscript.

**Response:** Thank you very much for your careful reviewing. The mistake was corrected in the revised manuscript.

“Among them, the repulsion of K363 and R348 in CAT significantly increases in the systems of DSB/ZnF1/ZnF3/CAT/Lig1 and DSB/ZnF1/ZnF3/CAT/Lig3.”
